# Tetra­chlorido­[*N* ^2^,*N* ^2′^-(di­methyl­silanedi­yl)bis­(*N*-*tert*-butyl-3-methyl­benzimid­amid­ato)-κ^2^ *N* ^2^,*N* ^2′^]hafnium(IV)

**DOI:** 10.1107/S1600536813030328

**Published:** 2013-11-13

**Authors:** Tao Wang, Jian-Ping Zhao, Sheng-Di Bai

**Affiliations:** a Institute of Applied Chemistry, Shanxi University, Taiyuan 030006, People’s Republic of China

## Abstract

The symmetric title mol­ecule, [Hf(C_26_H_40_N_4_Si)Cl_4_], lies about a twofold rotation axis. The Hf^IV^ and Si atoms lie on the rotation axis with all other atoms being in general positions. The Hf^IV^ atom is six-coordinated by two N atoms from the *N* ^2^,*N* ^2′^-(di­methyl­silanedi­yl)bis­(*N*-*tert*-butyl-3-methyl­benz­imid­amidate) ligand and four Cl^−^ ions in a slightly distorted octa­hedral geometry. The two amidinate moieties are connected through the central Si atom with Si—N bond length of 1.762 (3) Å, generating the characteristic N—C—N—Si—N—C—N skeleton of a silyl-linked *ansa*-bis­(amidine) species.

## Related literature

For reviews of related amidinate ligands and their applications, see: Edelmann (2012 ▶); Lei *et al.* (2011 ▶); Münch *et al.* (2008 ▶). For a review of the modification of the steric and electronic properties of amidinate ligands by varying their substitution patterns, see: Liu *et al.* (2013 ▶); Qian *et al.* (2010 ▶). For related silyl-linked bis(amidinate) ligands and the synthesis of their metal complexes, including a closely related Hf complex, see: Bai *et al.* (2013 ▶).
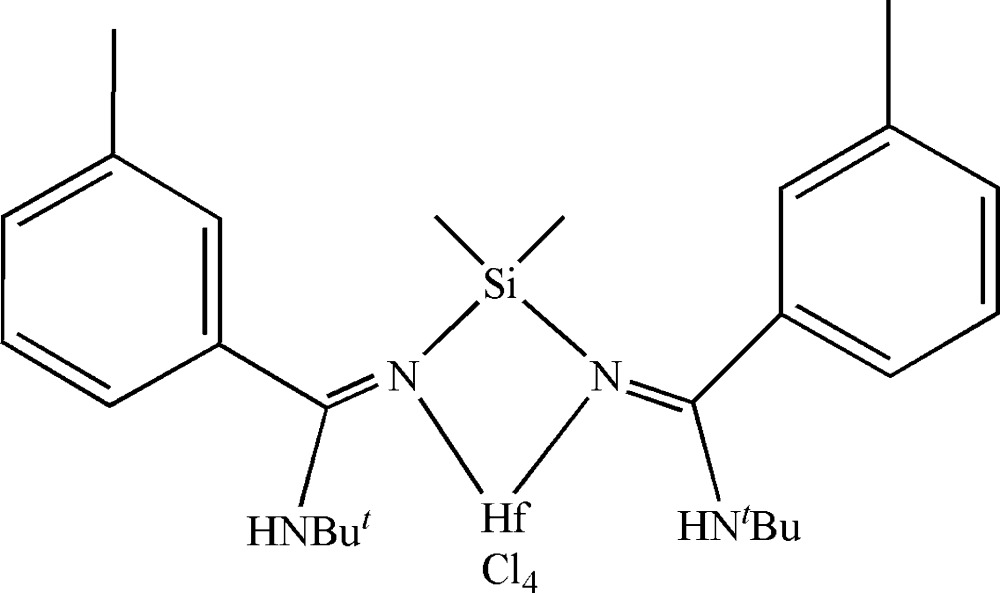


## Experimental

### 

#### Crystal data

[Hf(C_26_H_40_N_4_Si)Cl_4_]
*M* *_r_* = 757
Monoclinic, 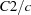

*a* = 9.4373 (14) Å
*b* = 17.992 (3) Å
*c* = 19.966 (3) Å
β = 103.276 (3)°
*V* = 3299.5 (8) Å^3^
*Z* = 4
Mo *K*α radiation
μ = 3.54 mm^−1^
*T* = 296 K
0.08 × 0.05 × 0.05 mm

#### Data collection

Bruker SMART area-detector diffractometer
Absorption correction: multi-scan (*SADABS*; Sheldrick, 1996 ▶) *T*_min_ = 0.765, *T*_max_ = 0.843
7121 measured reflections
2920 independent reflections
2446 reflections with *I* > 2σ(*I*)
*R*_int_ = 0.035

#### Refinement

*R*[*F* ^2^ > 2σ(*F* ^2^)] = 0.028
*wR*(*F* ^2^) = 0.061
*S* = 1.02
2920 reflections
169 parameters
H-atom parameters constrained
Δρ_max_ = 0.53 e Å^−3^
Δρ_min_ = −0.29 e Å^−3^

### 

Data collection: *SMART* (Bruker, 2000 ▶); cell refinement: *SAINT* (Bruker, 2000 ▶); data reduction: *SAINT*; program(s) used to solve structure: *SHELXS97* (Sheldrick, 2008 ▶); program(s) used to refine structure: *SHELXL97* (Sheldrick, 2008 ▶); molecular graphics: *SHELXTL/PC* (Sheldrick, 2008 ▶); software used to prepare material for publication: *SHELXL97*.

## Supplementary Material

Crystal structure: contains datablock(s) I, global. DOI: 10.1107/S1600536813030328/sj5366sup1.cif

Structure factors: contains datablock(s) I. DOI: 10.1107/S1600536813030328/sj5366Isup2.hkl

Additional supplementary materials:  crystallographic information; 3D view; checkCIF report

## Figures and Tables

**Table 1:** Selected bond lengths (Å)

Hf1—N2	2.233 (3)
Hf1—Cl2	2.4261 (11)
Hf1—Cl1	2.4366 (11)
Hf1—Si1	3.0588 (16)
Si1—N2	1.762 (3)
Si1—C13	1.857 (5)
N1—C5	1.318 (5)
N1—C1	1.504 (5)
N1—H1	0.8600

**Fig. 1.**
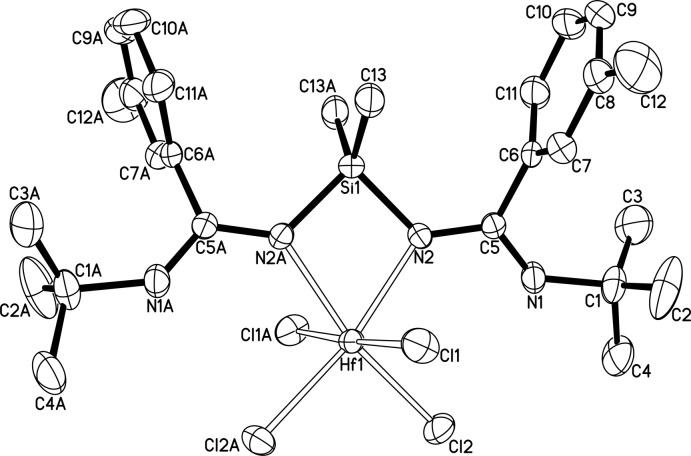
The molecular structure, showing the atom–numbering scheme. Displacement ellipsoids are drawn at the 30% probability level. Hydrogen atoms are omitted for clarity.

## supplementary crystallographic information

### 1. Comment

Anionic N,*N*-chelating amidinate ligands, have been widely used in the synthesis of organometallic complexes of the s-, *p*-, d-, and f-block metals for a number of years (Edelmann, 2012; Münch *et al.*, 2008). Their steric and electronic properties can easily be modified by a simple variation of the substitution pattern (Liu *et al.*, 2013; Qian *et al.*, 2010). In the search for ancillary ligands to replace cyclopentadienyls to create non-metallocene species, amidinate anions have found many applications in coordination chemistry, and also as ancillary ligands to form metal complexes which act as catalysts in organic transformations and ethylene polymerizations (Lei *et al.*, 2011).

Linked bis(amidinate) ligands are a very special branch of this class of compound and their chemistry has been developed in recent years. We explored a class of silyl linked bis(amidinate) ligands, and applied them to the synthesis of metal complexes. They imposed a close contact between the two amidinate moieties and had the advantage of affording binuclear complexes analogous to an "ansa-metallocene" (Bai *et al.*, 2013). Here, the synthesis and characterization of the Hf(IV) complex SiMe_2_[NC(*m*-MePh)N(Bu*^t^*)H]_2_HfCl_4_ bearing the silyl-linked ansa-bis(amidine) ligands will be described.

*N*',*N*''-(dimethylsilanediyl)bis(*N*-*tert*-butyl-3-methylbenzimidamide SiMe_2_[NC(*m*-MePh)NH(*^t^*Bu)]_2_ was prepared by treating *^t^*BuNH_2_ with one equivalent of LiBu*^n^*, *m*-MePhCN, and half equivalent of SiMe_2_Cl_2_ in a one-pot reaction. Treating the ansa-bis(amidine) SiMe_2_[NC(*m*-MePh)NH(*^t^*Bu)]_2_ with HfCl_4_ in CH_2_Cl_2_ gave the title compound. Crystals suitable for *X*–ray investigation were obtained by recrystallization from toluene and its molecular structure is presented in Fig. 1. It is a symmetric molecule lying about a 2-fold rotation axis (*e* in Wyckoff notation). The Hf1 and Si1 atoms lie on this axis with all other atoms on general positions. The two amidinate moieties connect the central Si atom with Si–N2 distances 1.762 (3) Å, which matched our original proposal of forming a dianionic N–C–N–Si–N–C–N framework. The structure shows that all the substituents and the silyl bridge are on the same side of the N—C—N skeletons, resulting in two E-anti forms of the amidinate units. The two inner nitrogen atoms bind to Hf1 at a distance of 2.233 (3) Å, and the N2—Hf2—N2^i^ angle is 69.28 (11)° (i = -*x* + 1, *y*, -*z* + 1/2). The Hf center also exhibits a slightly distorted octahedral geometry.

### 2. Experimental

A solution of LiBu*^n^* (2.2 *M*, 2.27 ml, 5.0 mmol) in hexane was added to a stirred solution of *^t^*BuNH_2_ (0.53 ml, 5.0 mmol) in THF (*ca* 30 ml) by syringe at 273 K. The reaction mixture was warmed to room temperature and kept stirring for 4 h and then *m*-MePhCN (0.59 ml, 5.0 mmol) was added by syringe at 273 K. The reaction mixture was warmed to room temperature and kept stirring for 4 h. Then SiMe_2_Cl_2_ (0.3 ml, 2.55 mmol) was added by syringe at 273 K. After stirring at room temperature for 4 h, it was dried in vacuum to remove all volatiles. The residue was extracted with CH_2_Cl_2_ (30 ml) and then HfCl_4_ (0.812 g, 2.5 mmol) was added to this stirred solution at 273 K. The reaction mixture was warmed to room temperature, after stirring for 4 h the solution was dried in vacuum to remove all volatiles. The residue was dissolved with toluene and then concentrated to yield colorless crystals of the title compound. Yield: 0.422 g (22.3%). ^1^H NMR (30 MHz, CDCl_3_) δ (p.p.m.): 8.813 (s, 2H; N*H*), 7.398, 7.204 (d, 8H; *m*-Me*phenyls*), 2.511 (s, 6H; *m*-*Me*phenyls), 1.034 (s, 18H; *^t^Bu*), -0.272 (s, 6H; Si*Me*_2_). ^13^C NMR (75 MHz, CDCl_3_) δ (p.p.m.): 172.566 (N-*C*-N), 139.174–126.831 (*m*-Me*phenyl*), 78.569–77.724 (*m*-*Me*phenyls), 57.109 (*C*Me_3_), 32.080, 22.363 (C*Me*_3_), 3.012 (Si*Me*_2_). Anal. Calcd. for C_26_H_40_Cl_4_HfN_4_Si (Mr = 757.00): C, 45.25; H, 5.33; N, 3.30%. Found: C, 45.56; H, 5.48; N, 3.44%.

### 3. Refinement

The methyl H atoms were constrained to an ideal geometry, with C—H distances of 0.96 Å and *U*_iso_(H) = 1.5*U*_eq_(C), but each group was allowed to rotate freely about its C–C and C–Si bonds. The amino H atoms were constrained with N—H distances of 0.86 Å and *U*_iso_(H) = 1.2*U*_eq_(N). The phenyl H atoms were placed in geometrically idealized positions and constrained to ride on their parent atoms, with C—H distances of 0.93 Å and *U*_iso_(H) = 1.2*U*_eq_(C).

### Crystal data

**Table:**

[Hf(C_26_H_40_N_4_Si)Cl_4_]	*F*(000) = 1512
*M**_r_* = 757	*D*_x_ = 1.524 Mg m^−^^3^
Monoclinic, *C*2/*c*	Mo *K*α radiation, λ = 0.71073 Å
Hall symbol: -C 2yc	Cell parameters from 2487 reflections
*a* = 9.4373 (14) Å	θ = 2.5–22.1°
*b* = 17.992 (3) Å	µ = 3.54 mm^−^^1^
*c* = 19.966 (3) Å	*T* = 296 K
β = 103.276 (3)°	Block, colorless
*V* = 3299.5 (8) Å^3^	0.08 × 0.05 × 0.05 mm
*Z* = 4	

### Data collection

**Table:**

Bruker SMART area-detector diffractometer	2920 independent reflections
Radiation source: fine-focus sealed tube	2446 reflections with *I* > 2σ(*I*)
Graphite monochromator	*R*_int_ = 0.035
φ and ω scans	θ_max_ = 25.1°, θ_min_ = 2.3°
Absorption correction: multi-scan (*SADABS*; Sheldrick, 1996)	*h* = −11→7
*T*_min_ = 0.765, *T*_max_ = 0.843	*k* = −21→19
7121 measured reflections	*l* = −23→23

### Refinement

**Table:**

Refinement on *F*^2^	Primary atom site location: structure-invariant direct methods
Least-squares matrix: full	Secondary atom site location: difference Fourier map
*R*[*F*^2^ > 2σ(*F*^2^)] = 0.028	Hydrogen site location: inferred from neighbouring sites
*wR*(*F*^2^) = 0.061	H-atom parameters constrained
*S* = 1.02	*w* = 1/[σ^2^(*F*_o_^2^) + (0.0266*P*)^2^] where *P* = (*F*_o_^2^ + 2*F*_c_^2^)/3
2920 reflections	(Δ/σ)_max_ = 0.001
169 parameters	Δρ_max_ = 0.53 e Å^−^^3^
0 restraints	Δρ_min_ = −0.29 e Å^−^^3^

### Special details

**Table:**

Geometry. All e.s.d.'s (except the e.s.d. in the dihedral angle between two l.s. planes) are estimated using the full covariance matrix. The cell e.s.d.'s are taken into account individually in the estimation of e.s.d.'s in distances, angles and torsion angles; correlations between e.s.d.'s in cell parameters are only used when they are defined by crystal symmetry. An approximate (isotropic) treatment of cell e.s.d.'s is used for estimating e.s.d.'s involving l.s. planes.
Refinement. Refinement of *F*^2^ against ALL reflections. The weighted *R*-factor *wR* and goodness of fit *S* are based on *F*^2^, conventional *R*-factors *R* are based on *F*, with *F* set to zero for negative *F*^2^. The threshold expression of *F*^2^ > σ(*F*^2^) is used only for calculating *R*-factors(gt) *etc*. and is not relevant to the choice of reflections for refinement. *R*-factors based on *F*^2^ are statistically about twice as large as those based on *F*, and *R*- factors based on ALL data will be even larger.

### Fractional atomic coordinates and isotropic or equivalent isotropic displacement parameters (Å^2^)

**Table:**

	*x*	*y*	*z*	*U*_iso_*/*U*_eq_	
Hf1	0.5000	0.540155 (12)	0.2500	0.03621 (10)	
Cl1	0.63951 (14)	0.53649 (6)	0.36889 (6)	0.0584 (3)	
Cl2	0.33063 (15)	0.63063 (6)	0.27686 (7)	0.0666 (4)	
Si1	0.5000	0.37014 (8)	0.2500	0.0430 (4)	
N1	0.2205 (4)	0.47999 (19)	0.33427 (18)	0.0510 (10)	
H1	0.2186	0.5176	0.3074	0.061*	
N2	0.3964 (3)	0.43808 (16)	0.28086 (16)	0.0363 (8)	
C1	0.1179 (5)	0.4877 (3)	0.3814 (2)	0.0589 (13)	
C2	0.2003 (7)	0.4884 (4)	0.4547 (3)	0.120 (3)	
H2A	0.2335	0.4390	0.4683	0.180*	
H2B	0.1379	0.5055	0.4833	0.180*	
H2C	0.2825	0.5210	0.4596	0.180*	
C3	0.0055 (7)	0.4271 (3)	0.3680 (4)	0.112 (3)	
H3A	−0.0378	0.4246	0.3196	0.168*	
H3B	−0.0685	0.4375	0.3926	0.168*	
H3C	0.0509	0.3804	0.3832	0.168*	
C4	0.0435 (7)	0.5621 (3)	0.3634 (3)	0.0891 (19)	
H4A	0.1146	0.6012	0.3732	0.134*	
H4B	−0.0279	0.5693	0.3901	0.134*	
H4C	−0.0033	0.5630	0.3153	0.134*	
C5	0.3128 (4)	0.4280 (2)	0.32526 (19)	0.0337 (9)	
C6	0.3272 (5)	0.3587 (2)	0.3673 (2)	0.0415 (10)	
C7	0.4307 (5)	0.3561 (2)	0.4283 (2)	0.0510 (11)	
H7	0.4860	0.3984	0.4429	0.061*	
C8	0.4556 (7)	0.2921 (3)	0.4691 (3)	0.0682 (15)	
C9	0.3727 (8)	0.2312 (3)	0.4451 (3)	0.089 (2)	
H9	0.3867	0.1879	0.4712	0.107*	
C10	0.2714 (8)	0.2312 (3)	0.3850 (3)	0.088 (2)	
H10	0.2182	0.1883	0.3706	0.105*	
C11	0.2455 (6)	0.2957 (2)	0.3440 (3)	0.0652 (14)	
H11	0.1760	0.2960	0.3026	0.078*	
C12	0.5689 (8)	0.2921 (4)	0.5360 (3)	0.112 (2)	
H12A	0.5769	0.2430	0.5554	0.167*	
H12B	0.5408	0.3264	0.5674	0.167*	
H12C	0.6611	0.3068	0.5276	0.167*	
C13	0.6240 (6)	0.3125 (3)	0.3154 (2)	0.0676 (15)	
H13A	0.6920	0.2872	0.2943	0.101*	
H13B	0.5681	0.2768	0.3340	0.101*	
H13C	0.6761	0.3439	0.3517	0.101*	

### Atomic displacement parameters (Å^2^)

**Table:**

	*U*^11^	*U*^22^	*U*^33^	*U*^12^	*U*^13^	*U*^23^
Hf1	0.04771 (16)	0.03029 (14)	0.03166 (14)	0.000	0.01126 (11)	0.000
Cl1	0.0724 (8)	0.0574 (7)	0.0391 (6)	−0.0115 (6)	−0.0002 (5)	0.0005 (5)
Cl2	0.0921 (10)	0.0479 (7)	0.0683 (8)	0.0238 (7)	0.0361 (8)	0.0044 (6)
Si1	0.0556 (11)	0.0302 (8)	0.0490 (11)	0.000	0.0241 (9)	0.000
N1	0.056 (2)	0.056 (2)	0.048 (2)	0.0136 (19)	0.0266 (19)	0.0120 (17)
N2	0.0409 (19)	0.0351 (17)	0.0345 (18)	−0.0027 (15)	0.0123 (16)	0.0020 (14)
C1	0.054 (3)	0.080 (3)	0.049 (3)	0.014 (3)	0.025 (2)	0.000 (2)
C2	0.109 (5)	0.207 (7)	0.044 (4)	0.072 (5)	0.018 (4)	−0.010 (4)
C3	0.096 (5)	0.095 (4)	0.174 (8)	−0.010 (4)	0.093 (5)	−0.014 (5)
C4	0.091 (4)	0.092 (4)	0.100 (5)	0.042 (4)	0.055 (4)	0.014 (3)
C5	0.035 (2)	0.037 (2)	0.028 (2)	−0.0086 (19)	0.0047 (18)	−0.0062 (16)
C6	0.058 (3)	0.036 (2)	0.039 (3)	−0.004 (2)	0.028 (2)	−0.0045 (18)
C7	0.061 (3)	0.047 (3)	0.048 (3)	0.000 (2)	0.020 (2)	0.005 (2)
C8	0.097 (4)	0.062 (3)	0.054 (3)	0.020 (3)	0.035 (3)	0.015 (3)
C9	0.174 (7)	0.041 (3)	0.076 (4)	0.008 (4)	0.076 (5)	0.011 (3)
C10	0.160 (7)	0.041 (3)	0.080 (4)	−0.033 (3)	0.062 (5)	−0.015 (3)
C11	0.085 (4)	0.058 (3)	0.061 (3)	−0.023 (3)	0.032 (3)	−0.013 (3)
C12	0.134 (6)	0.123 (5)	0.072 (5)	0.036 (5)	0.013 (4)	0.039 (4)
C13	0.084 (4)	0.062 (3)	0.071 (4)	0.030 (3)	0.045 (3)	0.025 (3)

### Geometric parameters (Å, º)

**Table:**

Hf1—N2	2.233 (3)	C3—H3C	0.9600
Hf1—N2^i^	2.233 (3)	C4—H4A	0.9600
Hf1—Cl2^i^	2.4261 (11)	C4—H4B	0.9600
Hf1—Cl2	2.4261 (11)	C4—H4C	0.9600
Hf1—Cl1^i^	2.4366 (11)	C5—C6	1.491 (5)
Hf1—Cl1	2.4366 (11)	C6—C7	1.377 (6)
Hf1—Si1	3.0588 (16)	C6—C11	1.390 (6)
Si1—N2^i^	1.762 (3)	C7—C8	1.399 (6)
Si1—N2	1.762 (3)	C7—H7	0.9300
Si1—C13^i^	1.857 (5)	C8—C9	1.368 (8)
Si1—C13	1.857 (5)	C8—C12	1.507 (8)
N1—C5	1.318 (5)	C9—C10	1.351 (8)
N1—C1	1.504 (5)	C9—H9	0.9300
N1—H1	0.8600	C10—C11	1.409 (7)
N2—C5	1.327 (5)	C10—H10	0.9300
C1—C2	1.491 (7)	C11—H11	0.9300
C1—C3	1.502 (7)	C12—H12A	0.9600
C1—C4	1.516 (7)	C12—H12B	0.9600
C2—H2A	0.9600	C12—H12C	0.9600
C2—H2B	0.9600	C13—H13A	0.9600
C2—H2C	0.9600	C13—H13B	0.9600
C3—H3A	0.9600	C13—H13C	0.9600
C3—H3B	0.9600		
			
N2—Hf1—N2^i^	69.32 (16)	H2A—C2—H2C	109.5
N2—Hf1—Cl2^i^	164.88 (9)	H2B—C2—H2C	109.5
N2^i^—Hf1—Cl2^i^	97.96 (9)	C1—C3—H3A	109.5
N2—Hf1—Cl2	97.96 (9)	C1—C3—H3B	109.5
N2^i^—Hf1—Cl2	164.88 (9)	H3A—C3—H3B	109.5
Cl2^i^—Hf1—Cl2	95.71 (6)	C1—C3—H3C	109.5
N2—Hf1—Cl1^i^	94.22 (9)	H3A—C3—H3C	109.5
N2^i^—Hf1—Cl1^i^	83.21 (9)	H3B—C3—H3C	109.5
Cl2^i^—Hf1—Cl1^i^	92.23 (4)	C1—C4—H4A	109.5
Cl2—Hf1—Cl1^i^	89.86 (4)	C1—C4—H4B	109.5
N2—Hf1—Cl1	83.21 (9)	H4A—C4—H4B	109.5
N2^i^—Hf1—Cl1	94.22 (9)	C1—C4—H4C	109.5
Cl2^i^—Hf1—Cl1	89.86 (4)	H4A—C4—H4C	109.5
Cl2—Hf1—Cl1	92.23 (4)	H4B—C4—H4C	109.5
Cl1^i^—Hf1—Cl1	176.89 (5)	N1—C5—N2	120.5 (3)
N2—Hf1—Si1	34.66 (8)	N1—C5—C6	119.6 (3)
N2^i^—Hf1—Si1	34.66 (8)	N2—C5—C6	119.9 (3)
Cl2^i^—Hf1—Si1	132.14 (3)	C7—C6—C11	119.6 (4)
Cl2—Hf1—Si1	132.14 (3)	C7—C6—C5	118.7 (4)
Cl1^i^—Hf1—Si1	88.45 (3)	C11—C6—C5	121.5 (4)
Cl1—Hf1—Si1	88.45 (3)	C6—C7—C8	122.2 (5)
N2^i^—Si1—N2	92.2 (2)	C6—C7—H7	118.9
N2^i^—Si1—C13^i^	116.87 (18)	C8—C7—H7	118.9
N2—Si1—C13^i^	108.80 (19)	C9—C8—C7	116.7 (5)
N2^i^—Si1—C13	108.80 (19)	C9—C8—C12	122.9 (5)
N2—Si1—C13	116.87 (18)	C7—C8—C12	120.4 (5)
C13^i^—Si1—C13	112.1 (3)	C10—C9—C8	122.8 (5)
N2^i^—Si1—Hf1	46.09 (10)	C10—C9—H9	118.6
N2—Si1—Hf1	46.09 (10)	C8—C9—H9	118.6
C13^i^—Si1—Hf1	123.93 (17)	C9—C10—C11	120.6 (5)
C13—Si1—Hf1	123.93 (17)	C9—C10—H10	119.7
C5—N1—C1	133.8 (4)	C11—C10—H10	119.7
C5—N1—H1	113.1	C6—C11—C10	117.9 (5)
C1—N1—H1	113.1	C6—C11—H11	121.0
C5—N2—Si1	127.0 (3)	C10—C11—H11	121.0
C5—N2—Hf1	131.4 (2)	C8—C12—H12A	109.5
Si1—N2—Hf1	99.25 (14)	C8—C12—H12B	109.5
C2—C1—C3	111.6 (5)	H12A—C12—H12B	109.5
C2—C1—N1	110.4 (4)	C8—C12—H12C	109.5
C3—C1—N1	110.6 (4)	H12A—C12—H12C	109.5
C2—C1—C4	109.5 (5)	H12B—C12—H12C	109.5
C3—C1—C4	109.2 (5)	Si1—C13—H13A	109.5
N1—C1—C4	105.2 (4)	Si1—C13—H13B	109.5
C1—C2—H2A	109.5	H13A—C13—H13B	109.5
C1—C2—H2B	109.5	Si1—C13—H13C	109.5
H2A—C2—H2B	109.5	H13A—C13—H13C	109.5
C1—C2—H2C	109.5	H13B—C13—H13C	109.5

Symmetry code: (i) −*x*+1, *y*, −*z*+1/2.
